# “My brain freezes and I am blocked again”: The subjective experience of post-migration living difficulties influenced by complex posttraumatic stress disorder of Afghan asylum seekers and refugees in Austria

**DOI:** 10.1371/journal.pone.0288691

**Published:** 2023-07-26

**Authors:** Jennifer Schiess-Jokanovic, Christine Gösling-Steirer, Viktoria Kantor, Matthias Knefel, Dina Weindl, Brigitte Lueger-Schuster

**Affiliations:** Department of Clinical and Health Psychology, Faculty of Psychology, University of Vienna, Vienna, Austria; Fondazione Policlinico Universitario Agostino Gemelli IRCCS, Universita’ Cattolica del Sacro Cuore, ITALY

## Abstract

**Background:**

Potentially traumatic experiences and post-migration living difficulties (PMLDs) undoubtedly leave marks on mental health and psychosocial functioning. While PMLDs are recognised as a risk factor for posttraumatic stress disorder and complex posttraumatic stress disorder (described together here as C/PTSD), recent investigations have found that C/PTSD symptoms might also influence the experience of PMLDs. The subjective experience of and coping with PMLDs in the context of C/PTSD symptoms has not yet been explored.

**Methods:**

Semi-structured, interpreter-assisted interviews exploring the subjective experience of post-migration living difficulties were conducted with treatment-seeking Afghan refugees and asylum seekers (N = 24) and transcribed verbatim. Participants were screened using the International Trauma Questionnaire (ITQ) and allocated to a C/PTSD group or non-C/PTSD group. We analysed the qualitative interviews using content analysis and then compared the results of the two groups.

**Results:**

Over half of the participants (58.3%) met the criteria for C/PTSD. While the two groups addressed numerous similar themes, the C/PTSD group more frequently mentioned themes associated with C/PTSD symptoms (e.g., intrusions, avoidance, sleep disturbances, affective dysregulation) that influenced their responses to PMLDs. The non-C/PTSD group more often experienced positive emotions such as gratitude and optimism, and showed more active, solution-oriented behaviour as well as positive self-verbalisation.

**Conclusion:**

To achieve a deeper understanding of PMLDs, post-traumatic psychopathology should be taken into account, as C/PTSD symptoms influence the experience of and coping with PMLDs. The specifics of individual experiences need to be considered in order to promote adaptive coping with PMLDs and to set individual trauma-focused and transdiagnostic treatment targets. In addition, psychological interventions should incorporate psychoeducation to improve the understanding of the impact of C/PTSD on the current experience of PMLDs.

## Introduction

Afghans represent the third largest refugee population in the world [1]. Since the Taliban takeover in 2021, the number of Afghan asylum seekers and refugees (ASR) has increased for the 15^th^ consecutive year [1]. The following year, Afghanistan was the most common country of origin of asylum seekers in Austria [2]. The Afghan population comprises over 45,000 individuals in Austria, making this ASR population one of the largest in this country [3]. Numerous asylum seekers and refugees (ASR) from Afghanistan have been affected by high rates of potentially traumatic experiences (PTEs) before and during their flight [4, 5] and also have to deal with a multitude of migration-specific stressors [6].

The construct of so-called post-migration living difficulties (PMLDs) includes intrapersonal (e.g., worries about the future, homesickness), interpersonal (e.g., isolation, conflicts), societal (e.g., discrimination, asylum law, mass media), and cultural stressors (e.g., culture, foreign language) [7–9]. Research has revealed that the impact of PMLDs on mental health problems and reduced quality of life is comparable to the impact of potentially traumatic experiences [4, 10].

Since the outbreak of the COVID-19 pandemic, socio-economic challenges and mental health problems have become increasingly common among the Austrian population [11, 12] and particularly among ASR in Austria, who already faced instability and crises before [13, 14]. Accordingly, ASR show a higher prevalence of mental health problems, which is higher still in treatment-seeking ASR populations [4, 15, 16]. Compared to ASR from other countries of origin, individuals from Afghanistan in Austria were found to have lower levels of education, longer waiting periods for asylum decisions, poorer health and lower quality of life [17–19].

Stress-related disorders such as posttraumatic stress disorder (PTSD) and complex posttraumatic stress disorder (CPTSD) are among the most commonly reported mental health problems among ASR in high-income countries [20, 21]. In the ICD-11, the diagnostic criteria for CPTSD encompass the symptom clusters of PTSD (re-experiencing, avoidance, sense of threat) and three additional symptom clusters associated with disturbances in self-organisation (affective dysregulation, negative self-concept, disturbances in relationships) as well as functional impairment [22]. Besides the general functional impairments associated with PTSD or CPTSD (C/PTSD) [23, 24], ASR with C/PTSD have been found to suffer from higher levels of distress associated with PMLDs [25, 26]. However, using the sum of PMLDs as predictor of psychopathological distress is problematic [27, 28], providing little insight into the underlying mechanisms of action and limited guidance for psychological therapy for ASR [29].

To better capture the complex interplay between PMLDs and mental health in ASR, ecological models have suggested mutual relationships between and within different forms of protective and risk factors (e.g., PMLDs) [7, 30, 31]. Moreover, according to these models, an experienced loss of resources might increase the risk of further loss and set in motion a spiral of loss [31]. For example, prior to migration, a variety of satisfying social relationships might stabilise a vulnerable self-esteem. After migration, traumatic experiences and a loss of social resources might contribute to a destabilisation of self-esteem and increase the risk of developing CPTSD. Low self-esteem might make it harder to establish new contacts in the host country and increase mental health problems, which may in turn further diminish self-esteem and exacerbate, for instance, social withdrawal, difficulties acquiring the language of the host country, and psychopathology.

In parallel to the postulated ecological models, research on various forms of psychopathology has investigated the complex interplay of individual symptoms [32], but has rarely included external factors [33, 34]. Accordingly, the interplay between different forms of PMLDs and psychopathology at the symptom level has rarely been examined so far. However, initial studies have reported associations beyond the constructs of PMLDs and psychopathology, supporting the assumption that individual symptoms might interact in distinct ways with different forms of PMLDs [29, 35–37]. Studies comparing the associations between PMLDs and anxiety disorders, depression, and PTSD have indicated the strongest associations between PTSD and almost all forms of PMLDs [26, 38]. Therefore, it seems especially important to examine this relationship in depth.

Further analyses of the associations between different forms of PMLDs and CPTSD symptom clusters have revealed an important role of re-experiencing and affective dysregulation [39]. This finding is consistent with previous evidence that people with C/PTSD use more maladaptive coping strategies such as rumination or avoidance [40, 41]. The recognition of these associations represents important information per se. However, the question of how CPTSD symptoms influence the subjective experience of and coping with PMLDs in daily life among ASR cannot be sufficiently investigated using checklist-based quantitative methods [42].

Initial qualitative investigations reported differences in the experience of PMLDs between ASR with low and high levels of PTSD symptoms [8, 43]. ASR with high PTSD showed lower levels of attachment to both the home and host country [8, 43] and had fewer future plans. Moreover, they experienced higher levels of distress, social isolation, relationship problems and conflict, used more avoidance-based strategies to deal with these problems [8] and reported poorer self-reflection [43]. In comparison, ASR with lower levels of PTSD were more likely to feel comfortable in their new community. They were pleased with their housing conditions and had sufficient money for their daily needs as well as access to health care services, education, skills training, and employment programs [8]. Furthermore, individuals with low PTSD reported a clearer sense of identity and subjective meaning in the refugee experience [43]. To the best of our knowledge, hardly any research has employed a qualitative approach to explore how ASR experience and cope with PMLDs while taking into account CPTSD symptoms beyond the classic symptoms of PTSD [8, 43].

To promote better mental health care for ASR in the future, the main aim of this study was to explore the subjective experience of and coping with PMLDs under consideration of C/PTSD symptoms. Based on previous empirical studies, we hypothesised that individuals with C/PTSD might differ from those without C/PTSD in terms of cognitive, emotional, physical and/or behavioural responses and coping strategies to PMLDs. The more frequent use of maladaptive coping strategies, such as rumination or avoidance, among ASR with C/PTSD was expected to contribute to increased distress related to PMLDs and potentially generate additional stressors. By conducting an in-depth examination of the differences in responses to PMLDs between ASR with and without C/PTSD, valuable insights might be gained into the underlying characteristics of these reactions and the influencing role of C/PTSD. Furthermore, we examined the subjectively experienced consequences of potentially traumatic experiences among Afghan ASR and their influence on daily life. These findings may offer initial guidance for the development of interventions that integrate approaches to reduce C/PTSD and address the distress due to PMLDs. By incorporating these insights into mental health care, we can strive for improved support and well-being for ASR in the future.

## Materials and methods

This study was part of the research project “A brief transdiagnostic psychological intervention for Afghan asylum seekers and refugees” (PIAAS-Study) [44, 45].

### Study procedure

The study was approved by the Ethics Committee of the University of Vienna (reference numbers: 00356 and 00445). A study protocol was published for quality assurance and to provide a detailed description of the study procedure [45]. Recruitment took place mainly in cooperation with two non-governmental organisations and outpatient treatment centres in Vienna, but also with numerous smaller cooperation partners and disseminators. The staff informed potential participants on the waiting list about the PIAAS-Study and handed out information material in Dari. If the person were interested, they had the possibility to personally contact the research team or the institution forwarded contact details with the consent of the persons. The research team then contacted the individuals and arranged appointments. Which were all conducted with a clinical psychologist trained in psychological counselling skills together with an interpreter. Regular supervision took place for quality assurance, cultural sensitivity, and prevention of risks to the mental health of the research team.

At baseline participants were provided with detailed information, and written informed consent was obtained. Subsequently, the demographic data and the PTEs were collected during the baseline assessment. C/PTSD symptoms were captured over the course of the baseline and post-assessment. Due to the high number of illiterate Afghan ASR, the German and Dari versions of the self-report questionnaires were administered in a fully structured interview format. The German-speaking psychologist read out the German version of each item and an interpreter read out the Dari equivalent. An adapted version of the transdiagnostic psychological intervention “Problem Management Plus” with a total of six 90-minute sessions was offered to the participants [44].

Following the intervention and the post-assessment, we contacted participants of the intervention arm (N = 26) and asked them to take part in a qualitative interview about their subjective experiences in Austria. In total, 24 (92.3%) individuals agreed to participate. Of the other two participants, one participant was not reachable and the other declined to participate without giving a reason. The qualitative interviews took place between November 2020 and March 2021. The initial semi-structured interviews were conducted face-to-face in the “Outpatient Unit Research, Teaching, and Practice” of the Faculty of Psychology at the University of Vienna, while subsequent interviews were conducted via telephone conference due to the COVID-19 lockdown in Austria beginning on the 17^th^ November 2020. The participants were asked to go to a quiet place where they could speak openly and undisturbed. All participants gave consent for the interview to be recorded for further analysis. Technical glitches or interruptions were rare and included brief connection problems, a one-time call interruption, and a one-time interruption by a child. Subsequently, all recordings were transcribed verbatim.

### Participants

Inclusion criteria for study participation were 1) treatment-seeking adult Afghan ASR, 2) Dari as native language, 3) elevated psychological distress on the Refugee Health Screener-15 (RHS-15) questionnaire (total score ≥ 12 or Distress Scale score ≥ 5), and 4) participants of the intervention arm after completed post-assessment. The exclusion criteria, which were observed and asked during the interview at the baseline assessment, were 1) severe cognitive impairment (moderate to severe intellectual disability or dementia) which hinders the necessary understanding of study-relevant contents and questions, 2) ongoing trauma-focused therapy, and 3) current physical or mental health conditions that require other treatment and/or interfere with participation (e.g., acute suicidality, psychotic symptoms). Each participant was assigned a pseudonym. An overview of the sociodemographic characteristics of the total sample (N = 24) is provided in Table 1.

**Table 1:** Sample characteristics for each group.

	C/PTSD (n = 15)	non-C/PTSD (n = 9)
Gender (female) n (%)	5 (33.3)	7 (77.8)
Age Mean (SD)	32.3 (12.9)	40 (14.2)
Marital status n (%)		
Single	7 (46.7)	1 (11.1)
Married	6 (40.0)	6 (66.7)
In a relationship	1 (6.7)	-
Divorced	1 (6.7)	1 (11.1)
Widowed	-	1 (11.1)
Asylum status n (%)		
Secure asylum status^a^	10 (66.7)	8 (88.9)
Insecure asylum status	5 (33.3)	1 (11.1)
Trauma events^b^ Mean (SD)		
Childhood trauma	6 (4.4)^c^	4.2 (6.2)
Adulthood trauma	7.6 (6.0)^c^	6.9 (4.3)

Note. N = 24.

^a^ secure asylum status includes unconditional residence permits with a long-term perspective (>1 year) and free access to the labour market in Austria.

^b^ Total of self-experiences PTEs

^c^ n = 14 due to missing data in the HTQ.

### Measures

C/PTSD was surveyed using the International Trauma Questionnaire (ITQ). The ITQ is an 18-item measure comprising six items on the PTSD subscale (symptom clusters of re-experiencing, avoidance, sense of current threat), and for CPTSD six additional items on the Disturbances in Self-Organisation subscale (DSO; symptom clusters of affective dysregulation, negative self-concept, disturbances in relationships). A further three items on each subscale measure functional impairment. The severity of C/PTSD symptoms was assessed referring to the last month using a five-point Likert scale (0 = "Not at all" to 4 = "Extremely"). The C/PTSD diagnosis was considered as fulfilled if a participant scored at least 2 (“moderately”) on one of the items in each symptom cluster and functional impairment. The ITQ has good psychometric properties [46]. In the current study the Cronbach’s alpha coefficient was .89.

PTEs were assessed using an adapted version of the Harvard Trauma Questionnaire (HTQ) Checklist with 29 dichotomous items (yes/no) [47]. The adaptations of the questionnaire included additional items and a specifier for childhood or adulthood trauma. The HTQ was developed and validated in ASR samples [48].

A semi-structured interview guide was developed [49, 50] to investigate the participants’ subjective experience of and coping with PMLDs. The interview guide comprised of a cover sheet for notes on the behavioural observation and included open questions. The qualitative interview consisted of three sections: 1) general questions about life in Austria, 2) exploration of the subjective experience and coping with significant experiences (e.g., PMLDs, daily stressors) in Austria as well as the meaning of traumatic experiences in daily life, and 3) experiences with the intervention offered. Only section 2 was relevant for this study. Further details on this section are provided in the interview guide (see S1 Table).

### Data analysis

All research steps were conducted in accordance with the consolidated criteria for reporting qualitative studies (COREQ) [51]. The open-ended questions were analysed using content-structuring qualitative content analysis (QCA) [52], which is a systematic, rule-based method of text analysis. At the beginning of the analysis, the main thematic categories were deductively derived from the interview guide. First, we coded the cognitive, emotional, physical and behavioural reactions to the reported experiences. Second, we coded the reported traumatic experiences and their subjective meaning for the participants’ life. In the next step, we used an inductive approach for the coding system. To increase the quality of the analysis, two of the authors (JSJ and CGS) coded the main thematic categories for the entire interviews separately. The inductive subcategories were developed in a collaborative discourse and were coded independently by JSJ and CGS. Coding was then compared and any discrepancies were discussed with the last author (BLS) until consensus was reached. Intra/inter-coder agreement testing took place once 20% of the individual coding was complete. The agreement lay at 87.67%, which is considered acceptable according to intercoder reliability guidelines [53]. MAXQDA 2020 [54] was used for data management and analysis. Following the coding of all material with the entire category system, the quantitative data were analysed for the group allocation using the recommended diagnostic scoring for C/PTSD [46]. Participants were assigned to the C/PTSD group if a diagnosis of PTSD or CPTSD was met at the baseline and/or post-assessment.

## Results

### Aim 1: Subjective experience of and coping with PMLDs

When asked to describe subjectively meaningful experiences in Austria, each participant reported between one and ten different experiences occurring individually or jointly (e.g. discrimination during a job interview). These 16 identified themes are presented in Table 2. The most frequently reported cognitive, emotional, physical and behavioural reactions to these experiences in a comparison between the C/PTSD group and the non-C/PTSD group are described in more detail below. For all themes and their corresponding frequencies, see Table 3.

**Table 2:** Themes related to post-migration living difficulties & daily stressors.

Major Themes	Themes	C/PTSD n (%)	non-C/PTSD n (%)
Post-migration living difficulties	Health service utilisation	9 (60.0)	6 (66.6)
	Occupation^a^	11 (73.3)	3 (33.3)
	Language barriers	5 (33.3)	6 (66.6)
	Discrimination	6 (40.0)	5 (55.5)
	Asylum procedure	6 (40.0)	3 (33.3)
	Initial time in Austria	5 (33.3)	4 (44.4)
	Basic needs^b^	7 (46.4)	2 (22.2)
Daily stressors	Daily duties	3 (20.0)	4 (44.4)
	Parenting	4 (26.6)	4 (44.4)
	Relationship problems	9 (60.0)	6 (66.6)
	COVID-19 pandemic	2 (13.3)	3 (33.3)
	Health problems	5 (33.3)	1 (11.1)
Anticipated stressors	Appointments^c^	5 (33.3)	4 (44.4)
	Interrogation (Asylum procedure)	3 (20.0)	1 (11.1)
	Social events	1 (6.6)	2 (22.2)
	Performance situations	1 (6.6)	1 (11.1)

*Note*. N = 24 (n = 15 for C/PTSD group, n = 9 for non-C/PTSD group).

^a^ Occupation is defined as commitment to training or work activities

^b^ Basic needs refer to housing or financial problems

^c^ Appointments include different types of dates (e.g., medical appointments or official channels).

**Table 3:** Themes related to reactions and coping strategies.

Major themes	Themes	Subthemes	C/PTSD n (%)	Non-C/PTSD n (%)
Cognitive reactions and coping strategies	Recurrent thoughts	Intrusive memories and rumination	12 (80.0)	6 (66.6)
		Worries about the future	9 (60.0)	6 (66.6)
		Thought avoidance	11 (73.3)	3 (33.3)
	Expectancies	Fulfilled expectations	10 (66.6)	4 (44.4)
		Disappointed expectations	12 (80.0)	5 (55.5)
	Staying present	Problems in staying present	8 (53.3)	3 (33.3)
	Self-verbalisation	Positive self-verbalisation	3 (20.0)	8 (88.8)
		Self-devaluation	5 (33.3)	2 (22.2)
	Evaluation of the situation	Reappraisal	3 (20.0)	6 (66.6)
		Acceptance	4 (26.6)	3 (33.3)
	Low self-esteem		1 (6.6)	5 (55.5)
	Development	Positive development	10 (66.6)	8 (88.8)
		Stagnation	6 (40.0)	3 (33.3)
	Suicidality		3 (20.0)	0 (0)
	Relationship with others	Identification with others	6 (40.0)	3 (33.3)
		Devaluation of others	5 (33.3)	3 (33.3)
		Independence	4 (26.6)	4 (44.4)
		Sufferance	7 (46.6)	6 (66.6)
Emotional reactions and complaints	Anxiety		7 (46.6)	8 (88.8)
	Gratitude		2 (13.3)	4 (44.4)
	Loneliness		2 (13.3)	1 (11.1)
	Depression		6 (40.0)	1 (11.1)
	Feeling safe		2 (13.3)	2 (22.2)
	Helplessness		4 (26.6)	1 (11.1)
	Optimism		2 (13.3)	4 (44.4)
	Shame, guilt		4 (26.6)	2 (22.2)
	Stress		11 (73.3)	7 (77.7)
	Sadness		9 (60.0)	5 (55.5)
	Anger		3 (20.0)	1 (11.1)
	Non-specific good feelings		5 (33.3)	4 (44.4)
	Non-specific bad feelings		11 (73.3)	5 (55.5)
Physical reactions and complaints	Sleep disturbances		8 (53.3)	3 (33.3)
	Stress-related somatic problems		2 (13.3)	2 (22.2)
	Physical restlessness		3 (20.0)	3 (33.3)
	Stress-related pain		7 (46.6)	3 (33.3)
	Body weakness		7 (46.6)	3 (33.3)
Behavioural reactions and coping strategies	Distraction		7 (46.6)	4 (44.4)
	Aggressive behaviour		5 (33.3)	1 (11.1)
	Substance use		5 (33.3)	1 (11.1)
	Supporting others		3 (20.0)	3 (33.3)
	Avoidance		9 (60.0)	5 (55.5)
	Active, solution-oriented behaviour		8 (53.3)	7 (77.7)
	Seeking and accepting social support		12 (80.0)	8 (88.8)
	Social withdrawal, inactivity		11 (73.3)	7 (77.7)

*Note*. N = 24 (n = 15 for C/PTSD, n = 9 for non-C/PTSD group).

### Cognitive reactions and coping strategies

Eight themes were identified in the analysis of reported cognitive responses and coping strategies. The most prominent themes were recurrent thoughts, expectancies, staying present, and development.

#### Recurrent thoughts

This theme was divided into three subthemes: intrusive memories & rumination, worries about the future, and thought avoidance. While both groups were concerned about the future, 80% of the C/PTSD group reported intrusive memories & rumination about traumatic or stressful experiences as well as unsuccessful attempts at thought avoidance to deal with these memories.

Intrusive memories & rumination were repeatedly triggered by a variety of events (e.g., discrimination, conversations with others). Some participants emphasised that intrusive memories & rumination were stronger during the night or during inactive days and were associated with negative emotionality. While the non-C/PTSD group was less likely to report intrusive memories, two participants of this group nevertheless reported infrequent intrusive memories, triggered for example by common conversations.

" . . .sometimes I hear someone say, "the Taliban killed my uncle or killed my brothers”, and then it all comes back. . . . Those memories come back. THAT all comes back and I think to myself that it is happening right now… that my husband is just being killed.” (Zahra, non-C/PTSD group, age 57)

Participants in both groups expressed many different worries about the future, referring to numerous issues (e.g., worries about relatives, uncertain future). Worries due to an insecure asylum status and fear of being deported to Afghanistan were mentioned more frequently in the C/PTSD group. "Life is not safe because of the residence permit. And because I don’t know if I can stay here or what will happen then." (Qualid, C/PTSD group, age 27)

In dealing with rumination and worries about the future, thought avoidance was mentioned by over 70% of the C/PTSD group but only 33.3% of the non-C/PTSD group. As one female participant stated, "It’s hard to forget the past. But I have to forget the past because otherwise it eats me up inside if I keep thinking about the past." (Nabila, non-C/PTSD group, age 59).

#### Expectancies

In the analysis of expectancies, two divergent subthemes, more frequently reported by the C/PTSD group, were identified: fulfilled and disappointed expectations about life in Austria. The first subtheme included, for example, the availability of education for oneself and one’s children, the subjective experience of safety due to protective laws and authorities, the importance of human rights, especially of women and children.

"Here, women have more rights, they are equal to men. In Afghanistan it is not like that. In Afghanistan women were without rights, especially when I was young. I was 13 years old when I was told that I had to marry this man." (Aliyah, C/PTSD group, age 54)

As an example of the second, contrasting subtheme, one participant stated that:

"You come to Europe to have a better life. I came to live a better life (sighs). But later you think to yourself—Okay, I’m 23 years old, I’m lying in bed and I am not able get up." (Bashir, non-C/PTSD group, age 26)

Numerous participants reported that plans had repeatedly failed. The lengthy asylum process and rejection of the asylum application experienced by some led to unbearable psychological distress and feelings of injustice. Further disappointed expectations were also associated with the inability to acquire the language, fulfil social roles or meet one’s own or others’ expectations.

#### Staying present

While mild confusion and poor concentration were reported by three individuals in the non-C/PTSD group, such as a less structured proceeding than usual when preparing for a social event or lack of concentration when studying. Over 50% of the C/PTSD group reported severe difficulties in staying present. Dissociative experiences occurred in stressful situations or when confronted with trauma-associated triggers.

"At some point in the middle of the interview (within the asylum procedure) I got lost. I mean by that I didn’t understand at all what the interpreter was saying or the judge was asking me. I didn’t know anything anymore at all." (Nasim, C/PTSD group, age 40)

Some participants shared their experiences of recurrent memory gaps, derealisation, and absentmindedness in everyday life.

#### Development

This theme was divided into the two subthemes positive developments and stagnation. Almost all participants in the non-C/PTSD group, but only two thirds of the C/PTSD group, reported positive developments over time. These included an improvement in physical and mental health. Important influencing factors were for example finding a job, acquiring the language, and improved coping strategies, which led to greater strength and self-confidence.

"I was finished at that time. I thought it was over. But now I have improved a lot. Because now I have a flat, I have a job. I’m taking a German course now. And I go to the gym and play football. I am EVERYWHERE now (laughs)." (Bashir, non-C/PTSD group, age 26)

However, negative or a lack of developments were reported several times in both groups. Some participants reported deteriorating mental and physical health and increasing challenges as well as lack of support in various areas.

"In Afghanistan, the difficulty was that there is war. You get killed . . . Yeah, you’re just done. But here it’s like you’re just breathing. And you get mentally and physically broken here. Here people (ASR) are like corpses." (Nasim, C/PTSD group, age 40)

### Emotional reactions

We identified a total of 13 different emotional reactions, which were reported with different levels of frequency between the two groups. While the non-C/PTSD group reported more positive emotions (e.g., hope, gratitude), the C/PTSD group more frequently experienced depression or non-specific bad feelings. The main four emotional reactions (stress, anxiety, sadness, and non-specific bad feelings) are discussed further in the following.

#### Stress

The majority of participants (>70%) in both groups reported stress in response to a variety of PMLDs (e.g., asylum procedures, search for housing, discrimination), physical pain, and anticipatory stress. One participant noted that stress increased in situations without an interpreter or on so-called ’bad days’, which were characterised by inactivity and rumination.

#### Anxiety

Similar to the above-mentioned stress reactions, almost all participants in the non-C/PTSD group (88.8%) reported anxiety, mostly associated with upcoming events. A few participants reported anxiety during their initial time in Austria, which usually passed after a short period. Nevertheless, one participant in this group reported seven years of persistent anxiety, “I was constantly afraid that I would get lost on the road (in the city). This fear sometimes . . . when I remember that now. I also get anxious again.” (Fatima, non-C/PTSD group, age 37). Of these, the anxiety mostly referred to existential issues such as insecure residency and financial problems in the C/PTSD group. Suddenly occurring anxiety without a definable reason was also reported.

#### Sadness

Participants in the C/PTSD group commonly reacted with sadness to various situations (e.g. discrimination), but also during overall "bad days." Participants in both groups reported sadness linked to intrusive memories and rumination, "And sometimes I even cry. Tears come to my eyes and even though I don’t want to think about it, it all comes back to my mind anyway." (Mustafa, C/PTSD group, age 57).

#### Non-specific bad feelings

Along with stress, non-specific bad feelings represented the most common emotional reaction in the C/PTSD group. This reaction was most often mentioned in the context of general life, but also arose over the course of the qualitative interview together with the impulse of avoidance. For instance, one participant reasoned, "I don’t feel comfortable thinking about it (traumatic experiences) or talking about it because then it still lingers. And I dwell on this story for some time afterwards." (Samira, C/PTSD group, age 36). In the non-C/PTSD group, non-specific bad feelings mainly occurred following concrete events (e.g., bad news from Afghanistan or discrimination).

### Physical reactions and complaints

Overall, few participants mentioned physical reactions and complaints compared to other types of reaction compared to other types of reactions. Only sleep disturbances were mentioned by more than 50% of the C/PTSD group.

#### Sleep disturbances

Sleep disturbances (e.g., nightmares) were reported more frequently by the C/PTSD group, with the distress induced by these disturbances often lasting until the next day. Only one participant from the non-C/PTSD group reported trauma-related nightmares, which had occurred directly after a recent terrorist attack in Vienna.

#### Stress-related pain

Stressful situations and persistent strain were linked to headaches as well as other types of pain in the interviewed participants. For example, following an unsuccessful search for accommodation, a participant who had been in a homeless shelter stated that “I had a lot of pain. You know, when I am worried or angry, the pain comes. I also said to my doctor: ‘This is pain that goes from my head to my back.’” (Karim, C/PTSD group, age 34).

### Behavioural reactions and coping strategies

ASR in the C/PTSD group were more likely to react with aggressive behaviour or substance use, whereas those in the non-C/PTSD group more frequently reported active, solution-oriented behaviour.

### Actively seeking and accepting support

Over 80% of the participants of both groups stated that they actively sought and/or accepted social support, or more rarely practical support, to cope with stressful situations. Social support by professionals, but also by family members or strangers provided the participants with confidence, reassurance, and encouragement. One participant described how a stranger actively intervened during a discrimination situation, "And there was this woman, and this old lady came and said what I would have wanted to say, and it felt very good." (Zaira, non-CPTSD group, age 40). Numerous participants also reported experiencing disappointment due to a lack of support or an inaccessibility of support due to language barriers.

#### Social withdrawal and inactivity

Across both groups, over 70% of participants reported social withdrawal and inactivity as a short-term response to their currently experienced event or when dealing with ongoing difficult situations or emotions. One participant recalled his behaviour when waiting for the asylum decision, "I spent five years in a room. I was only in this room. The wall . . . I have had no contact with anyone, it was very difficult. For five years!” (Nasim, CPTSD group, age 40).

#### Active, solution-oriented coping behaviour

About 80% of the non-C/PTSD group, but only about 50% of the C/PTSD group, reported active, solution-oriented coping behaviour. Both groups reported this kind of reaction to different experiences, but also when preparing for upcoming events. As an example, participants prepared for upcoming appointments or used active coping behaviour to reduce stress, such as breathing techniques. Several participants in the C/PTSD group predominantly actively sought training opportunities, a job or legal advice.

#### Avoidance

Over 50% of the participants of both groups reported avoiding stressful situations. For instance, some stopped searching for a job following repeated rejections, while others avoided social contact due to conflicts, unfulfilled family expectations, or simply because of emotional stress triggered by human contact. Some participants avoided conversations about the past, which triggered concentration problems or non-specific bad feelings.

### Aim 2: The subjective consequences of traumatic experiences for daily life

In the second part of the interview, the participants were asked about the subjective consequences of traumatic experiences for their daily life. While we did not specifically ask about traumatic experiences, they were mentioned repeatedly in the narratives and were therefore entered into the analysis. For the full list of identified themes, see Table 4.

**Table 4:** Themes related to traumatic experiences and subjective consequences of trauma.

Major Themes	Themes	C/PTSD n (%)	non-CPTSD n (%)
Traumatic experiences^a^	Murder of loved ones	2 (13.3)	1 (11.1)
	Danger during flight	6 (40.0)	5 (55.5)
	Intrafamilial trauma	6 (40.0)	2 (22.2)
	War-associated trauma	6 (40.0)	6 (66.6)
	Persecution	-	1 (11.1)
Subjective consequences of trauma	Societal consequences of war	1 (6.6)	4 (44.4)
	Mental and physical consequences	8 (53.3)	3 (33.3)
	Behavioural pattern	3 (20.0)	1 (11.1)
	Strength	1 (6.6)	2 (22.2)
	No consequences	3 (20.0)	3 (33.3)

*Note*. N = 24 (n = 15 for C/PTSD, n = 9 for non-C/PTSD group).

^a^ Traumatic experiences mentioned during the qualitative interview.

Most frequently, participants in the C/PTSD group (53.3%) reported individual psychological and physical consequences of potentially traumatic experiences, such as persistent anxiety despite now being in a safe environment. For instance, one participant reported, “That didn’t happen here, it all happened in Afghanistan, that’s where this pain I got came from." (Mustafa, C/PTSD group, age 57).

By contrast, participants in the non-C/PTSD group more often did not report personal consequences of PTEs, but rather societal consequences of war and flight for people from Afghanistan.

"I think that because there has been war in Afghanistan for so many years, for 30–40 years and so. They (Afghans) were all struggling before with the war, yes with this situation and so . . . But now, I think they are all psychologically burdened. And yeah, if just a little thing happens, they explode." (Fatima, non-C/PTSD group, age 37)

Fewer ASR reported emotional and behavioural patterns shaped by their past. Some described aggressive reactions, mistrust, avoidance of other people, and constantly caring for others while neglecting one’s own needs. One participant reported being constantly closed to others, "I can’t trust anyone anymore because the people I trusted betrayed me . . . I don’t talk about these things with anyone anyway." (Amar, C/PTSD Group, age 25). Several participants described positive consequences associated with a feeling of inner strength and increased self-confidence.

"After some time, I had to do something every day to somehow get ahead. Then I did that… I managed that. I said to myself, "I can do it on my own." And I started to feel stronger and stronger. " (Samira, C/PTSD group, age 36).

Overall, three participants from each group reported that they had not observed any consequences of potentially traumatic experiences, repeatedly answering this question simply with "no". One of the few explanations provided for this response was, "Because of the fact that I have come this far, I have not had any bad consequences." (Zaira, non-C/PTSD group, age 40).

## Discussion

The current study aimed to explore the role of C/PTSD symptoms in the subjective experience of and coping with PMLDs among Afghan ASR in Austria. The results revealed remarkable differences in the frequency of reported different types of PMLDs and associated reactions as well as coping strategies between ASR with and without C/PTSD. In particular, individuals with C/PTSD were more likely to report problems associated with unmet basic needs, occupation or asylum processes. Notably, distinct cognitive, emotional, physical, and behavioural responses were observed between the two groups. The C/PTSD group exhibited elevated levels of recurrent thoughts linked to PTEs and PMLDs. They also tended to employ cognitive and behavioural avoidance strategies more frequently. Moreover, individuals with C/PTSD reported more frequent and intense negative emotions, such as persistently depressed mood or pervasive non-specific bad feelings. Conversely, individuals without C/PTSD reported more frequent experiences of positive emotions, engaged in positive self-verbalisation, and perceived positive developments. It is noteworthy that despite recognizing numerous psychological and physical consequences of PTEs, few links have been made between these consequences of PTEs and their impact on perceptions of and responses to PMLDs.

Over half of the participants met the criteria for probable C/PTSD. In contrast to most previous research findings [17], a larger proportion of men met the criteria for probable C/PTSD in the present study. The majority of PMLDs reported by our participants reflected those that are already known from previous research [55–57]. Beyond the moderating effects of origin-related and country-specific factors [17, 55] as well as time spent in the host country [28], Afghan ASR with probable C/PTSD reported different types of PMLDs compared to those without. The lack of basic needs have previously been found to be particularly impactful among ASR and were associated with traumatic experiences and psychopathology [29]. In line with this, in the present study, Afghan ASR with probable C/PTSD were more likely to report unmet basic needs, occupational problems (e.g., unemployment), and an insecure asylum status. Along with previous research indicating a bidirectional relationship between PMLDs and psychopathology [36], we likewise suggest a reciprocal association. Many ASR experienced homelessness and financial hardship in their country of origin, and these potentially traumatic experiences might influence the subjective experience of unmet basic needs in the host country [28]. Indeed, in some cases PMLDs might act simultaneously as stressors and triggers [58]. Consistent with the assumption that not all forms of PMLDs have the same impact on mental health [28, 29], unmet basic needs might particularly exacerbate or even trigger C/PTSD symptoms. Moreover, the possibility to deal with these PMLDs might be further limited due to the insecure asylum status and associated prohibition to work, which was reported more frequently in ASR with probable C/PTSD.

Affective dysregulation is already recognised as a consequence of childhood trauma [59, 60] and is a criterion for a diagnosis of CPTSD in the ICD-11 [22]. In accordance with this, the present findings support the hypothesised differences in cognitive, emotional, physical and behavioural coping strategies between ASR with and without C/PTSD. In line with previous studies, our participants with probable C/PTSD reported a more frequent use of maladaptive coping strategies [40], which have been found to mediate the association of traumatic experiences with PTSD and with PMLDs [59, 61]. In contrast to previous research which found no associations between adaptive emotion regulation strategies and either traumatic experiences or PTSD [40, 59]), participants with probable non-C/PTSD more frequently reported adaptive coping strategies in the present study. Nevertheless, it should be critically noted that the relationship between the different coping strategies and mental health is predominantly ambiguous [41]. Therefore, to ascertain whether adaptive coping strategies actually have the desired effect, further in-depth research might be necessary.

In terms of cognitive responses and coping strategies, participants with probable C/PTSD frequently mentioned intrusive memories and rumination [62], which were accompanied by further cognitive, emotional, physical and behavioural reactions and coping strategies. Spiller et al. pointed out that fear reactivity, which is often increased in individuals with PTSD, might increase the frequency and intensity of intrusive memories and rumination [58]. In this vein, our participants reported thought avoidance and trying to forget traumatic experiences, which is a commonly used strategy to cope with triggers and distress among ASR [63, 64]. In line with previous research [65], about half of the ASR with probable C/PTSD in the present study described difficulties in staying present due to dissociative reactions or concentration problems, often related to rumination. Since dissociative symptoms, rumination and concentration problems are not included in the ICD-11 CPTSD diagnosis [22], few studies have examined these symptoms in individuals with CPTSD [65]. Overall, only three participants with probable C/PTSD in our study reported using reappraisal or positive self-verbalisation. Moreover, general positive aspects of life in Austria or positive personal developments were often mentioned only briefly by participants with probable C/PTSD, followed by more detailed reports of unmet expectations. By contrast, participants in the non-C/PTSD group reported concrete positive personal developments more frequently and in greater detail. We suggest that the high degree of stress might hinder positive adaptation while also increasing the likelihood of attentional bias, which might in turn reduce the perception of positive experiences and developments [66].

Several group differences emerged regarding emotional reactions and complaints. Overall, increased negative and reduced positive affectivity were associated with various psychopathologies, including PTSD [67]. Consistent with this finding, participants in the non-C/PTSD group more frequently reported positive emotions (e.g., gratitude, hope). The ASR with probable C/PTSD were more likely to report suffering from depression and non-specific bad feelings, which may indicate a lack of emotional clarity [68]. Many of the participants reported social withdrawal and inactivity in dealing their emotions as they did not believe they could change their own negative mood [69]. The resulting intense, persistent emotional reactions might point to an under-regulation of emotional hyperactivation already associated with C/PTSD [70].

Poor physical health has frequently been associated with C/PTSD [24]. Similar to findings from a study in Syrian refugees in Germany [71], sleep disturbances were the most commonly reported physical reaction and complaint among Afghan ASR in the present study. Indeed, over half of the ASR with probable C/PTSD reported sleep disturbances, which is unsurprising given that trauma-associated nightmares are a symptom of C/PTSD. As in other studies [72, 73], several participants reported increased stress and C/PTSD symptoms the day after suffering from nightmares. Additionally, in line with previous evidence [73], body weakness and stress-associated pain were also reported more frequently by the ASR with probable C/PTSD. These physical complaints, which often occurred spontaneously in response to PMLDs or were reported as chronic stressors, were associated with severe distress and, in rare cases, with suicidality.

In accordance with previous findings, the most commonly reported behavioural reactions and coping strategies in both groups were seeking and accepting social support, followed by social withdrawal and inactivity [8, 41, 74, 75]. Although social support has predominantly been considered as a protective factor, this strategy was repeatedly linked to increased mental health problems. In some cases, it remained unclear whether social support actually brought about emotional relief or a sense of connectedness. For instance, several participants addressed the psychosocial costs of social support, such as feeling pressure due to expectations or critique, in turn potentially leading to subsequent social withdrawal [41]. C/PTSD symptoms might lead to limited subjective experiences of interpersonal connectedness and social support [76]. Social withdrawal, inactivity and avoidance are often used to deal with negative emotions triggered, for example, by trauma-associated memories or PMLDs, and have been assumed to be interrelated with psychosocial distress (e.g., depression, rumination) [41]. Active, solution-oriented behaviour was reported less frequently by the ASR with probable C/PTSD [61]. Previous research has reported a positive relationship between lack of access to adaptive emotion regulation strategies and PTSD [68]. As emphasised by Perez et al. [77], it remains unclear whether access to adaptive emotion regulation strategies is actually lacking or whether this phenomenon may be explained by the reduced self-efficacy associated with childhood trauma. Nevertheless, the lack of active, solution-oriented coping strategies might hinder the successful handling of PMLDs and intensify emerging problems [78].

Many of our participants reported subjective consequences of traumatic experiences for their daily life, mostly comprising physical or psychological complaints [79]. While none of the participants described C/PTSD and its influence on the experience of PMLDs as a result of traumatic experiences [80], intrusive memories were repeatedly mentioned. Overall, few subjective consequences or traumatic experiences were reported.

### Strengths and limitations

The key strength of the present study lies in the examination of the role of C/PTSD symptoms in the subjective experience of PMLDs in a hard-to-reach, vulnerable treatment-seeking sample of traumatised Afghan ASR in Austria. The qualitative interviews allowed for a deeper understanding of the complex interplay between C/PTSD symptoms and PMLDs. Nevertheless, some limitations must be considered. One important limitation of the study is the limited generalisability of findings. Due to the small sample and the nature of the qualitative study, the in-depth understanding may not be applicable to the entire ASR population. Regional differences within or between countries can influence the prevalence, nature, perception, and handling of PMLDs among subpopulations of ASR. These variations should be considered for ASR from different countries of origin as well as different host countries. The sample included only participants from the intervention arm of a larger project, who had completed a psychological intervention and post-assessment. An influence of the psychological intervention can be assumed, as the interventions offered included strategies for calming down and problem solving and were thus used more often in the recent past than otherwise (44). The participants of this study proactively sought psychological therapy, indicating a potentially more receptive sample experiencing less stigma towards mental health problems. Although interpreter-assisted interviews enabled the participation of persons with low German-language skills, misunderstandings, socially desirable responding, and a loss of information in the translation process cannot be ruled out. Future studies might consider direct assessment (e.g., ecological momentary assessments; EMAs) of PMLDs and their psychosocial consequences in daily life. To increase the generalisability of the findings, it is recommended for future research to include larger samples of non-treatment-seeking Afghan ASR from different regions as well as to consider gender differences and comorbidities.

## Conclusions

This study contributes to the growing body of knowledge on the influence of C/PTSD on the experience of and coping with PMLDs. C/PTSD influences the frequency of and responses to PMLDs among ASR. The symptoms associated with C/PTSD, such as recurrent thoughts and avoidance, not only exhibit an association to PTEs, but also exert a profound influence on the experienced distress and the effective coping with PMLDs. The findings highlight the importance of considering PMLDs in treatment approaches for ASR with C/PTSD and might support mental health practitioners and other professionals to develop targeted interventions on different dimensions to promote mental health and psychosocial adaptation among ASR.

On the intrapersonal dimension, a consideration of the influence of C/PTSD on the experience of and coping with PMLDs might provide important clues for individual therapy targets (e.g., reducing rumination or avoidance, promoting active, solution-oriented coping behaviour). A better understanding of the consequences of trauma and of the relationship between psychopathology and PMLDs should be addressed through psychoeducation [71, 81].

Interventions on the interpersonal dimension should focus on reducing social isolation and enhancing connectedness. Community-based interventions, group therapy and participation in interest groups should be encouraged [71].

On the societal dimension, measures should be taken to promote cultural and trauma sensitivity among professionals (e.g., mental health practitioners, teachers). Language barriers should be reduced to facilitate social inclusion and access to health services for ASR. Low-threshold services, increased use of (online) interpreters and the offer of trauma-sensitive language courses might be helpful in this regard [82]. Support in finding accommodation and employment might help overcome barriers such as opaque bureaucracies and discrimination.

Measures on the cultural dimension predominantly include subjects that policy makers have not yet sufficiently addressed. In addition to providing funding for the aforementioned interventions, it is especially important to establish accelerated and transparent asylum procedures [71, 83].

## Supporting information

S1 Table Interview guide is available online. (DOCX)

## Abbreviations

ASR: asylum seekers and refugees
CPTSD: complex posttraumatic stress disorder
C/PTSD: CPTSD and/ or PTSD
DSO: disturbances in self-organisation
HTQ: Harvard Trauma Questionnaire Checklist
ITQ: International Trauma Questionnaire
PMLDs: post-migration living difficulties
PTSD: posttraumatic stress disorder
QCA: Qualitative Content Analysis
