# Supplementary material for: “My brain freezes and I am blocked again”: The subjective experience of post-migration living difficulties influenced by complex posttraumatic stress disorder of Afghan asylum seekers and refugees in Austria
Source: PLoS One. 2023 Jul 26;18(7):e0288691. doi: 10.1371/journal.pone.0288691 (PMC10370748; doi:10.1371/journal.pone.0288691)
Supplement: S1 Table — (DOCX) [file pone.0288691.s001.docx]

# Supporting information:

***My brain freezes and I am blocked again:* The subjective experience of post-migration living difficulties influenced by complex posttraumatic stress disorder**

Jennifer Schiess-Jokanovic^*a^, Christine Gössling-Steirer, Viktoria Kantor^a^, Matthias Knefel^a^, Dina Weindl^a^, Ingo Schäfer^b^, and Brigitte Lueger-Schuster^a^

Author Note

^a^ Department of Clinical and Health Psychology, Faculty of Psychology, University of Vienna, Vienna, Austria.

^b^ Department of Psychiatry and Psychotherapy, University Medical Centre Hamburg-Eppendorf, Hamburg, Germany.

*Correspondence concerning this article should be addressed to Jennifer Schiess-Jokanovic, Department of Clinical and Health Psychology, Faculty of Psychology, University of Vienna, Wächtergasse 1, 1010 Vienna, Austria. E-Mail: [jennifer.schiess-jokanovic@univie.ac.at](mailto:jennifer.schiess-jokanovic@univie.ac.at)

Table S1

*Interview guide*

| **Items** |
| --- |
| - I would be interested in hearing about experiences from your life in Austria, things that you often experience as a refugee. Could you tell me about such an experience, maybe something you have experienced recently?   - Are there also other experiences that you often have... bad ones? (if something positive was reported; otherwise go on to question   - (if no interpersonal situations were reported) And are there also experiences with other people? (possibly: Are there experiences that were similar but particularly bad for you?)   - Optional questions for a more detailed exploration of the situation (for questions 7-9, respectively): e.g.: Can you tell me a bit more about this? What did you do in the situation? How did you feel in this situation? Could you have done anything else? How did you feel after the situation? How long did the situation continue to bother you? How do you feel about it today? How often do such situations occur? Sometimes you know that a stressful situation is coming up in the near future. - How do you usually feel BEFORE stressful or difficult situations, when you know that such a situation is coming soon?   - Optional questions for a more detailed exploration of the situation: Do you have an example for me? How did you feel then? What did you do there? Would there have been other possibilities? Were there thoughts that preoccupied you? How long did you think about it? Does this often happen, that you get under pressure before such situations? - How do your traumatic experiences from the past influence your life in Austria today?   - Do the experiences from the past have an influence on your relationships with other people? Do they have an influence on other areas of your life? |
